# Tailored exercise with telehealth monitoring improves adherence and global health in kidney transplant recipients

**DOI:** 10.3389/fspor.2024.1436742

**Published:** 2024-09-03

**Authors:** Marco Vecchiato, Federica Duregon, Emanuele Zanardo, Veronica Baioccato, Giulia Quinto, Alberto Livio, Barbara Mazzucato, Chiara Sarri, Lia Bellis, Claudia Carella, Massimo Cardillo, Daniel Neunhaeuserer, Andrea Ermolao, Francesca Battista

**Affiliations:** ^1^ Sports and Exercise Medicine Division, Department of Medicine, University of Padova, Padova, Italy; ^2^ Clinical Network of Sports and Exercise Medicine of the Veneto Region, Veneto, Italy; ^3^ Centro Nazionale Trapianti, Istituto Superiore di Sanità, Rome, Italy

**Keywords:** KTR, exercise, physical activity, transplantation, telemedicine, device, tele-health

## Abstract

**Introduction:**

Tailored exercise prescription is a crucial intervention for kidney transplant recipients (KTRs). This longitudinal study investigates the impact on long-term effectiveness of exercise prescriptions over one year follow-up, implementing telehealth tools for exercise administration and adherence monitoring.

**Materials and methods:**

KTRs were evaluated with clinical assessments including body composition, blood and urinary parameters, physical performance and quality of life at baseline (T0), after six (T6) and twelve (T12) months. The adherence to prescribed exercise training was monitored via video call interviews until T6 when the sample was divided into a group monitored via wearables (WG) and a group continuing video calls (VG) until T12.

**Results:**

Twenty-six KTRs completed the study. No changes in body composition and kidney function were reported. KTRs showed an improvement in lipid profile, systolic blood pressure, cardiorespiratory fitness and quality of life. WG showed no clinical differences compared to VG except for reported higher quality of life.

**Discussion:**

A good adherence to the exercise prescription was obtained with both monitoring methods (232 vs 253 min/week). This study reinforces the inclusion exercise training for KTRs to enhance physical fitness and reduce cardiovascular risk factors. These results emphasize the role of telehealth monitoring methods as motivators for adherence to long-term exercise prescriptions.

## 1 Introduction

Kidney transplantation has significantly improved the survival and quality of life for individuals with end-stage kidney diseases. Despite advancements in medical care for kidney transplant recipients (KTRs), the risk of mortality from cardiac events remains significantly elevated, with an annual rate of fatal or nonfatal cardiovascular events up to 50 times higher compared to the general population (1, 2). This heightened cardiovascular risk is attributed to pre-existing conditions like hypertension and dyslipidemia, compounded by the effects of immunosuppressive therapy designed to prevent organ rejection (3).

Physical activity and exercise training have been demonstrated to counteract these mechanisms in patients with chronic kidney diseases and KTRs, mitigating inflammation, lowering blood pressure and cholesterol, and reducing endothelial disfunction (4, 5). For this reason, tailored exercise prescription has emerged in recent years as a promising intervention to address the physical deconditioning, enhance the functional capacity and improve overall quality of life of KTRs (6–8). Nevertheless, while exercise interventions are recognized as beneficial for global health, only few available studies have reported the effectiveness of this intervention over a prolonged period (9–11). Moreover, patient adherence to prescribed exercise is a critical concern, particularly in the context of KTRs, where routine exercise programs are not yet seamlessly integrated into standard clinical care. Telehealth tools to implement physical activity and exercise programs have been utilized in multiple studies due to their sustained benefits over time (12), with good results in increasing physical activity, improving body composition and fitness, across various age groups and both clinical and non-clinical populations (13), but poorly in KTRs and never to specifically monitor the efficiency and adherence to prescribed exercise (14, 15).

This longitudinal study primarily aims to investigate the impact of tailored exercise prescription and training on global health of KTRs over a one-year period with telehealth instruments implementation. A secondary aim is to verify the best method for improving adherence to exercise prescription.

## 2 Materials and methods

### 2.1 Participants

The study is part of a project sponsored by the Italian National Institute for Insurance against Accidents at Work (INAIL) to promote a multidisciplinary network and web applications to facilitate the resumption of an active lifestyle and the reintegration into the work and social activities for KTRs. This study included patients aged 18–65 years old who had undergone kidney transplantation at least six months before the first assessment and agreed to participate in a 12-month monitoring study including adherence to exercise prescription. Participants with any acute or chronic medical conditions that contraindicate high-intensity exercise or cardiopulmonary exercise testing (CPET) as severe cardiovascular, respiratory, or musculoskeletal disorders, were excluded from the study. Others exclusion criteria were physical and cognitive limitations that impeded exercise testing and evidence of acute transplant rejection at the time of enrollment. Recruitment took place from October 2020 through May 2022 at the Sports and Exercise Medicine Division of the University Hospital of Padova. The study was coordinated by the National Transplantation Centre and approved by the Ethics Committee of the Italian National Institute of Health with the code PRE BIO CE n.16146, 24/07/2019 and by the Local Ethic Committee with the code 4777/AO/19 - AOP1893 - BRIC 01, 22/01/2020. Written informed consent was obtained by the patients before inclusion, according to the procedures approved by the Ethics Committee in compliance with the Helsinki Declaration and national rules regarding clinical trial management.

### 2.2 Assessments

After eligibility evaluation, participants underwent a comprehensive global assessment including four different domains: body composition, blood and urinary parameters, physical performance and quality of life. During the monitoring period, the only changes in drug regimen were of immunosuppressive therapy.

#### 2.2.1 Body composition

Waist circumference was measured wrapping the measuring tape at the narrowest part of the abdomen, stand in front of the subject, between the iliac crest and the lowest rib. The body composition analysis was conducted using bioelectrical impedance analysis (Akern Srl, Florence, Italy). Impedance was measured through electrodes placed on the right hand and right foot of the participant in resting condition and under controlled room temperature and consistent hydration status. The resistance and reactance data acquired by the device were used to determine fat mass and fat-free mass using Bodygram 1.31 (Akern Srl, Florence, Italy).

#### 2.2.2 Blood and urinary parameters

Within one week prior to each assessment, patients underwent comprehensive blood and urine analyses, including the determination of kidney function, complete blood count, lipid profile, fasting glucose, C-reactive protein, and 24-h proteinuria. Standardized procedures were employed for the collection of blood and urine samples, conducted in the early morning following an overnight fasting period. Furthermore, patients refrained from engaging in exercise within the 48 h preceding samples collection.

#### 2.2.3 Physical performance

Maximal 12-leads ECG-monitored CPET (Masterscreen CPX system Jaeger, Carefusion, Hoechberg, Germany) on a cycle-ergometer (Cycle-ergometer eBike, General Electrics) or a treadmill (T170 DE-med, h/p/cosmos, Nussdorf-Traunstein, Germany) was performed, with an individually adapted test protocol in accordance with international guidelines until patients reached a Borg rating of perceived exertion (RPE) ≥ 18/20 (16, 17). Systolic and diastolic blood pressure (SBP and DBP) were measured at rest, every three minutes during exercise until peak and during the recovery phase. Continuous monitoring of the electrocardiogram was performed throughout the test and the respiratory gas exchange were monitored breath by breath during the whole test. Peak oxygen uptake (VO_2_ peak) was defined as the highest value of VO_2_ attained in a 30-s interval at peak exercise. The first ventilatory threshold (VT1) was identified on the plots of the cardiopulmonary evaluation using the simplified V-Slope method (17) while the second ventilatory threshold (VT2) was identified by an increase of the ventilatory equivalent for carbon dioxide and a decrease in end-tidal pressure of carbon dioxide (18).

Dominant and non-dominant handgrip strength was measured with a calibrated dynamometer (Baseline, Elmsford, NY, USA). Grip handle was adjusted if required and the elbow was flexed to 90° to guarantee the strongest grip strength measurement. For the analysis, an average of three standardized measurements was considered. The absolute handgrip strength values were then normalized for gender and body mass index (BMI) (19).

To assess lower limb strength, the 30 s Chair Stand Test (30CST), a component of the Senior Fitness Test battery, was employed. This test involves performing as many repetitions as possible within a predetermined 30-s period of rising from and sitting down on a chair. The starting position is seated with the upper limbs crossed over the chest, and a repetition is counted when the individual rises, completes the extension of the hip joint, and returns to the seated position (20).

The physical activity level was investigated with the International Physical Activity Questionnaire (IPAQ). This instrument is composed by a comprehensive set of domains including leisure time, domestic and gardening (yard) activities, work-related and transport-related activity. The items are structured to provide separate scores on walking, moderate-intensity, and vigorous-intensity activity, as well as a combined total score, expressed in MET/minutes/week, to describe overall level of activity (21).

#### 2.2.4 Quality of life

Short Form 36 (SF−36) questionnaire estimates physical and mental health through thirty-six questions across eight categories. The results can then be summarised in two summary scales:

- Physical component summary (PCS) including physical functioning, bodily pain, general health perceptions and physical role functioning.
- Mental component summary (MCS) including vitality, emotional role functioning, social role functioning, mental health or emotional wellbeing.

The scores for each domain can vary between 0 and 100, where 100 represents the best possible perception of quality of life (22).

**Figure 1:**
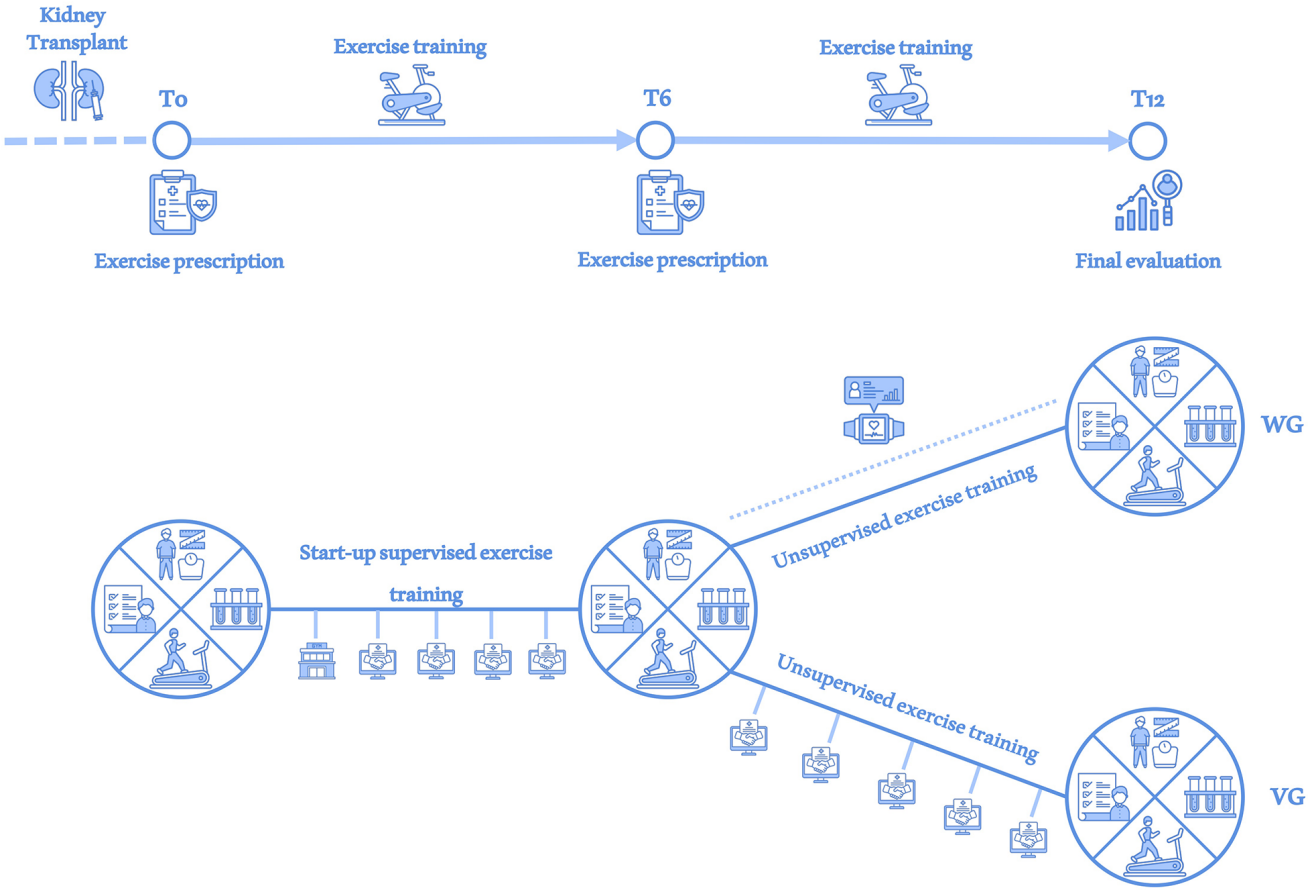
Study overview. At each assessment (T0, T6 and T12), investigations of the body composition data, blood and urinary parameters, physical performance and quality of life were performed (large circle divided into 4 sections). After a supervised exercise period during the first month, the entire study sample was monitored via monthly videocall interviews (handshake in the screen icon) until T6. At T6 the sample was divided into a WG group receiving physical activity monitoring via wearable device (smartwatch icon) until T12 and a VG group that continued to receive monitoring via videocall interviews until T12.

These assessments were conducted at baseline (T0), after six months (T6) and after twelve months (T12), as reported in Figure 1.

### 2.3 Exercise prescription

Based on the functional evaluation, a tailored exercise program was prescribed for each patient by a sports and exercise medicine physician, and all participants were invited to take part to a start-up supervised exercise training twice weekly, lasted one month and conducted by an exercise professional in the hospital gym. Exercise programs were tailored according to individual needs, particularly addressing any issues such as osteoarticular problems. Consequently, the intensity, volume, or type of exercises were adjusted to better meet each participant's requirements. Exercises targeted all major muscle groups, including squats, calf raises, seated rows, chest presses, and pull-downs, but were tailored for each participant based on their specific needs or limitations. Aerobic exercise intensities have been set according to the CPET results: - low intensity, under VT1; - moderate intensity, between VT1 and VT2; - high intensity, above VT2; as suggested by our working group and following international guidelines for exercise prescription (23, 24). Heart rates and RPE at VT1 and VT2 have been included in each patient's exercise prescription to clarify exercise intensity recommendations. Strength and flexibility exercise was planned based on previously described functional tests and following a standardized outline comprising breathing exercises and joint mobility warm-up at the beginning of the session, taking care not to exceed the level of discomfort. When possible, submaximal tests (10-Repetition Maximum) were also performed to determine the indicated percentage of intensity for each machine used. Where not possible, intensity in resistance training was based on RPE. The central part of the training comprised aerobic, resistance, proprioception and flexibility exercises targeted all major muscle groups. The prescribed exercise intensity was monitored using HR and the RPE scale, maintaining values corresponding to moderate intensity determined through CPET. Moreover, participants were encouraged to practice aerobic activity sessions independently; in this way, they were able to increase their weekly physical activity volume. Subsequently, at the end of the start-up training, exercise program was performed at home without a supervision, maintaining the same parts mentioned above (aerobic, resistance, flexibility training). At T6 adjustments in exercise prescriptions were made by the Sports and Exercise Medicine physician, as necessary, particularly regarding heart rates for aerobic exercise intensity; if no changes were needed, the initial T0 values were maintained. Further changes in exercise modalities have been implemented depending on the patient's clinical condition as already described for previous works of our group with serial CPETs (25).

### 2.4 Telehealth and monitoring methods

Between T0 and T6, after the supervised one-month start-up period in the hospital gym, the patients were monitored for adherence by monthly video interviews conducted by exercise professionals via a user-free dedicated platform. These video interviews aimed to investigate adherence with the exercise prescription, the amount of at least moderate physical activity performed per week, the frequency of structured exercise sessions conducted, and any clinical or exercise-related problems.

At T6 the included sample has been casually divided in two subgroups, without a standardized randomization. Smartwatches to monitor physical activity (Fitbit Versa 4, Fitbit Inc., California, USA) were randomly assigned to the half of participants who formed the Wearable Group (WG), ensuring an unbiased distribution for sex, age and self-reported physical activity level across the study population. KTRs who did not receive the wearable device continued to receive the monthly video interviews and formed the videocall group (VG), as shown in Figure 1. Heart rate thresholds to determine exercise intensities (low, moderate and vigorous) were configured by exercise professionals based on the parameters individuated during CPET. Patients were instructed to wear the device as much as possible throughout the day to collect data on step count, exercise duration and intensity. After acquiring informed consent from all WG patients, data collected were monitored and analysed through Fitabase platform (Fitabase, San Diego, California, USA), a cloud-based data aggregation platform to extract and aggregate data from devices.

### 2.5 Statistical analysis

Continuous variables were described using means and standard deviations and categorical variables were described using absolute values (*n*) and percentages (%). The Shapiro-Wilk test was used as distribution test. Comparisons between the same population at T0, T6 and T12 were performed with the Friedman test and *post hoc* analysis with Bonferroni correction. The WG and VG groups at T12 were compared for differences between the variables at T6-T12 with Mann Whitney U test. A *p*-value of <0.05 was considered statistically significant. Analyses were carried out using SPSS software (IBM SPSS Statistics for Windows, Version 26.0. Armonk, NY, USA: IBM Corp).

## 3 Results

### 3.1 Study sample

**Figure 2:**
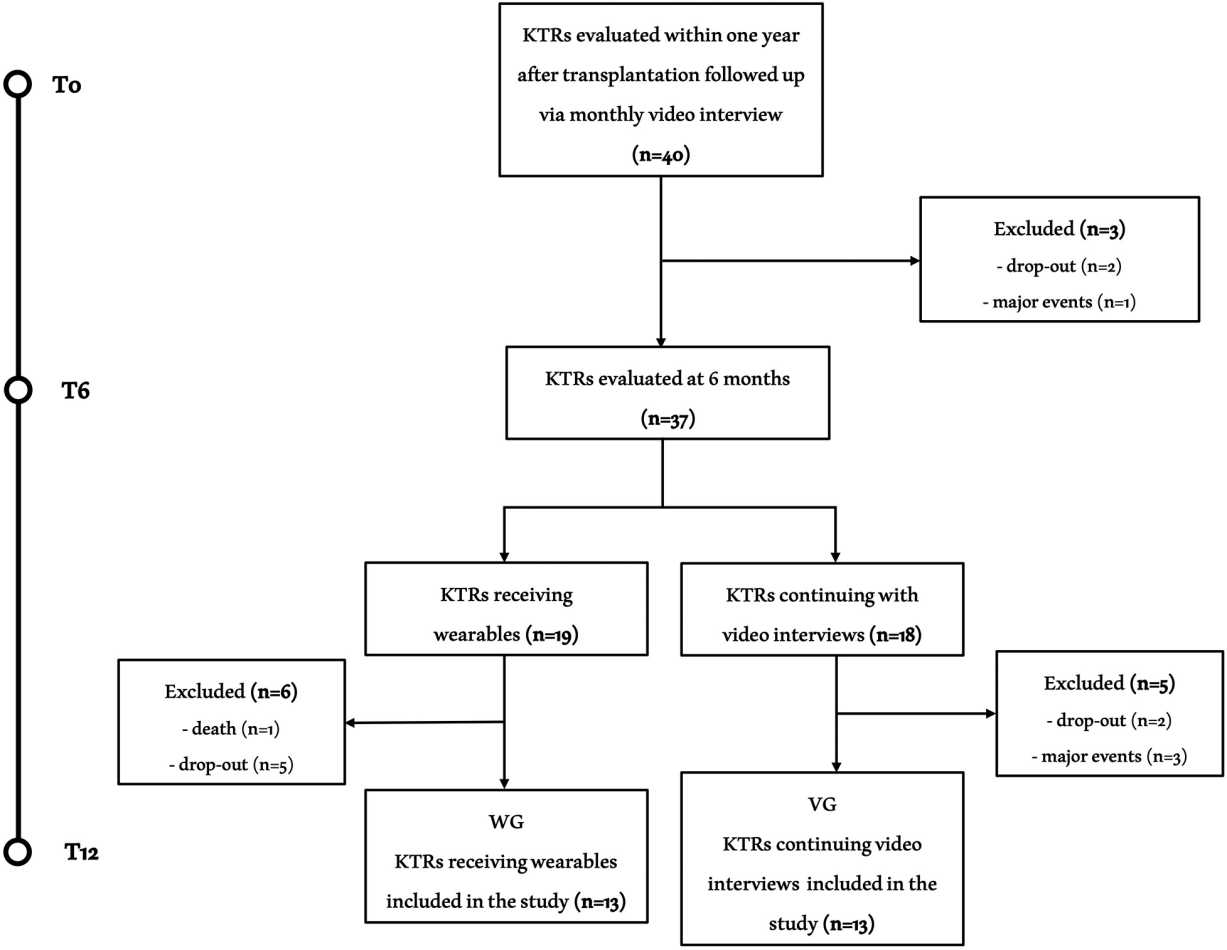
Study flow chart. Flow chart of the study. Of the 40 KTRs initially included, 26 completed all three assessments. The causes of exclusion were nine dropouts, four relevant clinical events (stroke, organ rejection, acute coronary artery disease and inguinal hernia with related inability to exercise) and one death (acute leukemia).

Of the 40 initials patients enrolled in the study, 26 KTRs successfully completed the entire exercise intervention period and were included in the final analysis (Figure 2). Clinical characteristics of the included sample and the causes for kidney transplantation were reported in Table 1. The mean age was 42.1 ± 9.0 years with 14 males and 12 females. The global evaluations performed on KTRs who completed all three assessments at T0, T6 and T12 are shown in Table 2.

**Table 1:** Baseline clinical characteristics of study participants at T0.

Clinical characteristics (*n* = 26)	
Age (years)	42.12 ± 9.04
Gender (males)	14 (53.85%)
Weight (kg)	65.75 ± 11.45
Height (cm)	166.69 ± 10.94
BMI (kg/m^2^)	23.54 ± 3.09
Pre-transplant dialysis time (months)	35.88 ± 61.67
Post-transplant time (months)	36.81 ± 22.72
Current smoking	1 (3.85%)
Dyslipidaemia under treatment	8 (30.77%)
Diabetes mellitus under treatment	2 (7.69%)
Hypertension under treatment	18 (69.23%)
Beta-blocker usage	12 (46.15%)
Immunosuppressive therapy:	26 (100%)
Tacrolimus	26 (100%)
Steroid therapy	23 (88.5%)
Mycophenolic acid	23 (88.5%)
Everolimus	2 (7.7%)
Pathologies leading to kidney transplantation:	
Polycystic kidney disease	8 (30.8%)
Unknown end-stage kidney disease	4 (15.6%)
Lupus nephritis	3 (11.6%)
Medullary sponge kidney	2 (7.7%)
Severe vesicoureteral reflux	2 (7.7%)
Wilms tumour	1 (3.8%)
Cystic fibrosis	1 (3.8%)
Diabetic kidney disease	1 (3.8%)
IgA nephropathy	1 (3.8%)
Bilateral kidney hypoplasia	1 (3.8%)
Tubulo-interstitial nephritis	1 (3.8%)
Iatrogenic kidney failure induced by mesalazine	1 (3.8%)

Data are expressed as mean ± standard deviation for quantitative variable or number (percentage) for qualitative variables.

**Table 2:** Complete assessments at T0, T6 and T12.

Parameters	T0	T6	T12	*p*
Body composition data				
BMI (kg/m^2^)	23.54 ± 3.09	23.66 ± 3.15	24.06 ± 3.31	0.407
Waist circumference (cm)	87.75 ± 12.46	87.52 ± 10.08	87.77 ± 9.71	0.455
Fat mass (%)	21.88 ± 5.09	21.86 ± 5.02	22.43 ± 5.09	0.176
Blood and urinary parameters				
Haemoglobin (g/L)	129.62 ± 20.17	130.69 ± 19.11	126.61 ± 30.05	0.280
Creatinine (mg/dl)	1.49 ± 0.52	1.46 ± 0.51	1.44 ± 0.51	0.417
Urea (mmol/L)	11.10 ± 5.61	13.05 ± 8.95	12.42 ± 8.21	0.957
eGFR (ml/min/1.73 m^2^)	56.96 ± 20.85	56.57 ± 19.19	54.69 ± 18.81	0.875
24-hour proteinuria (g)	0.28 ± 0.31	0.40 ± 0.41	0.31 ± 0.25	0.529
Total cholesterol (mg/dl)	212.88 ± 44.46	202.08 ± 40.82	203.77 ± 49.76	0.402
HDL-C (mg/dl)	55.50 ± 14.60	58.15 ± 12.88	60.42 ± 12.59	0.003*
LDL-C (mg/dl)	124.27 ± 37.47	112.24 ± 31.76	109.38 ± 33.71	0.446
Triglycerides (mg/dl)	151.64 ± 57.65	158.42 ± 67.13	169.81 ± 138.33	0.690
Glucose (mg/dl)	95.73 ± 14.64	97.73 ± 33.35	96.43 ± 25.05	0.131
Physical performance				
SBP rest (mmHg)	126.92 ± 11.67	123.08 ± 14.43	121.20 ± 13.48	0.018*
DBP rest (mmHg)	78.85 ± 11.25	75.96 ± 7.880	77.40 ± 9.37	0.761
SBP max (mmHg)	168.27 ± 19.90	169.04 ± 26.08	168.27 ± 21.86	0.796
DBP max (mmHg)	75.96 ± 16.73	73.08 ± 14.15	76.73 ± 13.11	0.730
VO_2_ max (ml/min)	1,694.74 ± 456.96	1,751.12 ± 464.42	1,842.17 ± 517.26	0.015*
VO_2_ max/kg (ml/kg/min)	25.71 ± 4.91	26.45 ± 4.86	27.41 ± 5.69	0.030*
HR VT1 (% of HR max)	73.51 ± 9.45	72.28 ± 9.43	72.66 ± 11.21	0.368
HR VT2 (% of HR max)	83.61 ± 19.07	87.26 ± 11.54	88.35 ± 12.38	0.352
HR max (bpm)	150.35 ± 17.76	147.42 ± 17.15	150.35 ± 18.70	0.123
Handgrip dominant limb (kg)	36.06 ± 12.00	38.23 ± 11.93	36.93 ± 11.79	0.346
Handgrip non dominant limb (kg)	34.77 ± 12.52	35.37 ± 10.70	33.85 ± 10.37	0.162
30CST (reps)	15.34 ± 4.12	17.27 ± 3.75	18.35 ± 4.70	0.003*°
IPAQ (METs/week)	1,619.61 ± 1,206.23	2,454.54 ± 1,386.08	2,908.23 ± 1,357.76	<0.001*°^§^
Quality of life				
MCS	60.10 ± 17.74	66.89 ± 16.48	69.20 ± 18.39	0.003*°
PCS	67.24 ± 19.94	74.15 ± 16.72	74.82 ± 16.41	0.001*°

BMI, body mass index; eGFR, estimated Glomerular Filtration Rate; H/LDL-C, high/low-density lipoprotein cholesterol; SBP, systolic arterial pressure; DBP, diastolic arterial pressure; VO_2_ max, maximal oxygen consumption; VT1, first ventilatory threshold; VT2, second ventilatory threshold; HR, heart rate; 30CST, 30-s chair stand; reps, repetitions; IPAQ, International Physical Activity Questionnaire; METs, metabolic equivalents of task; MSC, mental component summary; PCS, physical component summary.

*p* < 0.05 indicates a statistically significant difference.

*Post hoc* analysis was performed with Bonferroni correction: *: T0 vs. T12; °: T0 vs. T6; ^§^: T6 vs. T12.

### 3.2 Body composition data and haemato-urinary parameters

Comparing baseline body composition parameters with one year follow up values, no significant changes in BMI, waist circumference and fat mass were found. There were no differences in blood and urinary kidney functional values during the monitored period. Regarding the lipid profile, there was an improvement in HDL-cholesterol (from 55.50 to 60.42 mg/dl) and a non-significant decrease in LDL-cholesterol (from 124.27 to 109.38 mg/dl) with the total cholesterol value unchanged over the study period. No change in the glucose profile was detected. Three patients had C-reactive protein values compatible with inflammatory status at T0 (3.80 ± 1.35 mg/dl) and none at T6 and T12.

### 3.3 Physical performance and quality of life

SBP at rest showed a significant decline at one year follow-up. SBP and DBP at exercise peak showed no significant changes. CPET revealed significant enhancements in both absolute and relative VO_2_ peak among participants. No included patients presented symptoms, desaturation, or direct/indirect stress-inducible ischaemia signs during CPET. No changes in handgrip strength have been observed at T6 and T12 compared to T0, while the performance in the 30CST showed improvement at 6 months that was maintained at 12 months. IPAQ showed increases at both T6 and T12 (1,619.61 vs. 2,454.53 vs. 2,908.23 METs/week, *p* = 0.001).

SF-36 MCS and PCS showed improvement at T6 and a maintenance at T12 (*p* = 0.003 and *p* = 0.001, respectively).

### 3.4 Monitoring methodologies and adherence

During T0-T6 period the referred volume of moderate/vigorous physical activity in all study sample was 245.91 ± 22.81 min per week. At T6, out of the 19 KTRs receiving the wearable device, 13 patients completed the third assessment at T12 and were include in WG, while of the remaining 18 KTRs continuing with monthly videocall interviews, 13 completes the study protocol and composed the VG (Figure 2). There were no differences in gender (both groups had seven men and six women) or age (WG: 39.85 ± 9.34 vs. VG: 44.38 ± 8.47 years; *p* = 0.223) between the groups.

**Figure 3:**
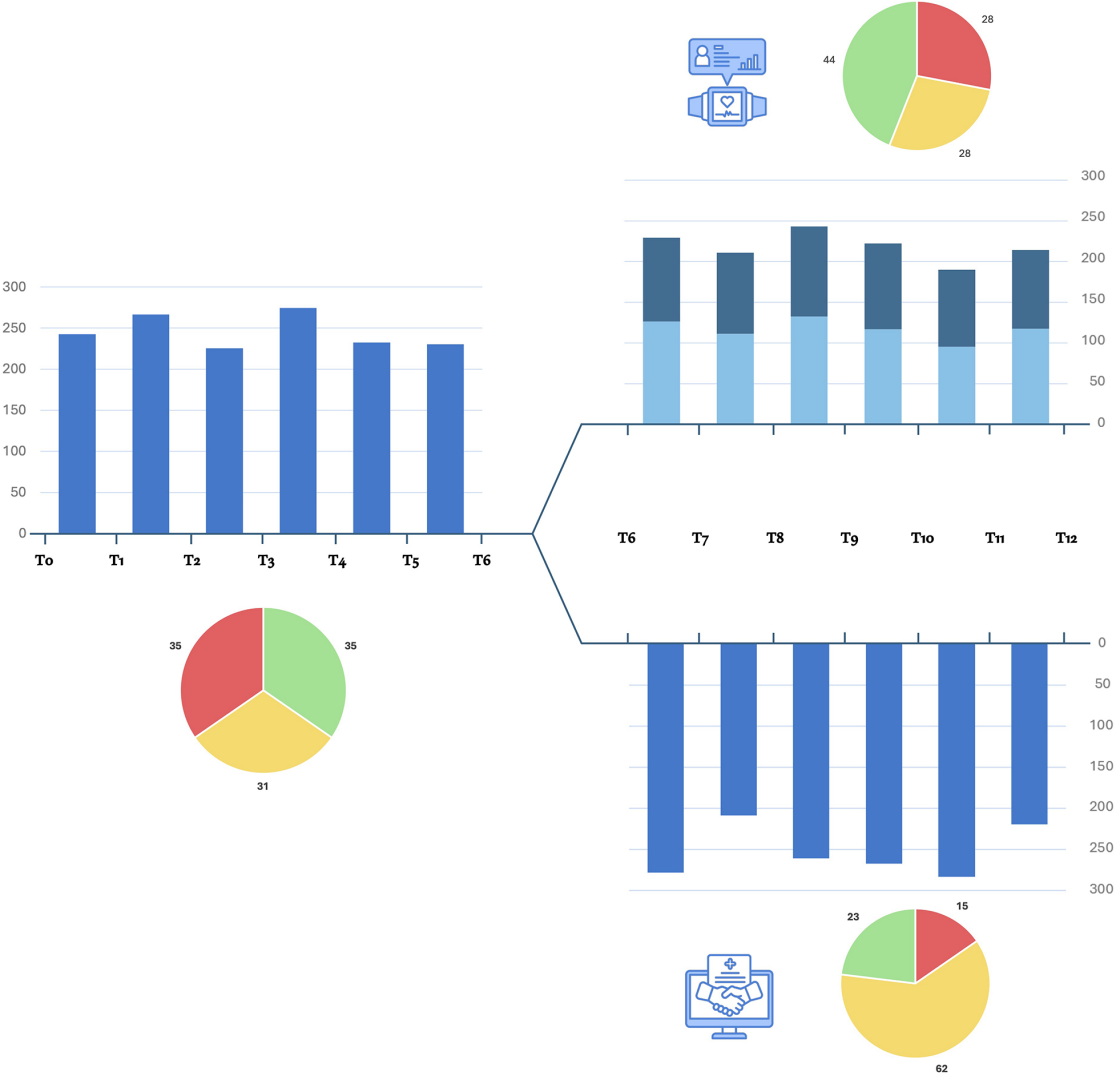
Monitoring of physical activity during the study. Blue bars show the average monthly level of moderate/vigorous activity per week (expressed as minutes per week) self-reported by video interviews during T0-T6 period and for the VG during T6-T12 period. For WG, moderate (light blue) and vigorous (dark blue) average monthly registered minutes of physical activity are shown as distinct. The cake diagrams show the percentage of patients who met the required minutes per week of moderate/vigorous physical activity: red, <150 min/week; yellow, 150–300 min/week; green, >300 min/week.

Among WG, continuous monitoring showed consistent engagement in physical activity and exercise training throughout the study period in 9 of the 13 patients received wearable monitoring with an average number of days of use of 143.38 ± 51.10. The recorded volume of moderate/vigorous physical activity was 231.98 ± 28.44 min per week with 9,584.47 ± 4,286.81 number of steps per day reached. Among VG, 11 of 13 patients self-reported regular physical activity throughout T6-T12 with an average of 1.42 ± 0.35 exercise sessions per week. Eight out of 13 patients reported mixed aerobic and strength activity and three reported predominantly aerobic activity. The average referred volume of moderate/vigorous physical activity was 252.85 ± 31.35 min per week. Four patients reported issues in the videocall interviews (two patients reported SBP increases, one patient reported musculoskeletal impairments in the upper limbs and one in the lower limbs), which resulted in an adaptation of the planned training schedule. The average data of physical activity level monitored during the study were shown in Figure 3.

No statistically significant differences in body composition, blood and urinary markers, and functional parameters were shown between the WG and VG. WG participants showed a higher value on the MCS (*p* = 0.003) and PCS (*p* = 0.010) compared to VG, as reported in Table 3.

**Table 3:** Comparison between WG and VG from T6 to T12.

Δ Parameters T6-T12	WGT6	WGT12	ΔWG	VGT6	VGT12	ΔVG	*p*
Body composition data							
BMI (kg/m^2^)	24.01 ± 3.55	24.40 ± 3.62	0.39 ± 2.63	23.26 ± 3.00	23.63 ± 3.23	0.40 ± 4.17	0.724
Fat mass (%)	21.28 ± 4.87	22.43 ± 5.58	1.15 ± 3.58	23.53 ± 5.75	23.30 ± 5.40	−0.23 ± 1.91	0.482
Blood and urinary parameters							
Creatinine (mg/dl)	1.52 ± 0.41	1.46 ± 0.49	−0.06 ± 0.54	1.42 ± 0.61	1.42 ± 0.55	0.00 ± 0.30	0.287
Urea (mmol/L)	12.19 ± 7.53	13.46 ± 9.70	1.27 ± 11.55	11.27 ± 6.67	11.54 ± 7.00	0.27 ± 10.04	0.311
Total cholesterol (mg/dl)	202.46 ± 34.65	210.31 ± 58.76	7.85 ± 36.30	201.69 ± 47.65	197.23 ± 40.17	- 4.46 ± 23.62	0.762
HDL-C (mg/dl)	55.00 ± 10.34	55.77 ± 7.53	0.77 ± 6.73	61.31 ± 14.73	65.08 ± 15.05	3.77 ± 6.13	0.479
Physical performance							
VO_2_ max (ml/min)	1,783.08 ± 527.74	1,861.72 ± 573.95	78.70 ± 110.72	1,719.15 ± 410.66	1,822.16 ± 476.62	103.49 ± 139.65	0.448
VO_2_ max/kg (ml/kg/min)	27.35 ± 4.77	28.24 ± 5.67	0.89 ± 2.09	25.55 ± 4.97	26.57 ± 5.81	1.02 ± 2.86	0.511
Handgrip dominant limb (kg)	38.47 ± 12.54	35.66 ± 11.69	−3.01 ± 4.17	41.06 ± 12.49	38.83 ± 12.47	−1.42 ± 4.67	0.186
30CST (reps)	16.62 ± 4.09	17.77 ± 4.87	1.15 ± 2.16	17.92 ± 3.40	18.92 ± 4.68	1.00 ± 3.39	0.999
IPAQ (METs/week)	2,015.64 ± 965.82	2,601.12 ± 1,307.02	585.47 ± 633.57	2,893.41 ± 1,628.62	3,215.37 ± 1,388.51	321.92 ± 543.35	0.448
Quality of life							
MCS	65.23 ± 12.44	72.49 ± 15.99	7.27 ± 12.02	68.56 ± 20.12	65.91 ± 20.62	−2.64 ± 4.60	0.009
PCS	70.08 ± 13.24	72.52 ± 14.66	2.44 ± 14.51	80.52 ± 15.55	79.43 ± 15.79	−1.09 ± 5.20	0.003
Physical activity monitoring and adherence							
Moderate physical activity (min/week)	126.56 ± 91.85	117.69 ± 103.29	−8.87 ± 72.27	245.45 ± 178.40	219.38 ± 162.93	−26.08 ± 67.64	0.854*
Vigorous physical activity (min/week)	102.83 ± 74.36	96.65 ± 84.82	−6.18 ± 81.70				

BMI, body mass index; HDL-C, high-density lipoprotein cholesterol; VO_2_ max, maximal oxygen consumption; 30CST, 30-s chair stand; reps, repetitions; IPAQ, International Physical Activity Questionnaire; METs, metabolic equivalents of task; MSC, mental component summary; PCS, physical component summary. The *p*-values reported refer to the difference in the parameters described between ΔWG and ΔVG.

* For this *p*-value the weekly amount of moderate and vigorous physical activity has been added for the ΔWG.

## 4 Discussion

Findings from the current study contribute to the growing body of evidence supporting the benefits of exercise prescription and interventions in KTRs. The observed improvements in the lipid profile, blood pressure and cardiorespiratory fitness underscore the potential of exercise as an integral component of post-transplant care. In addition, both monitoring methods allowed us to assess an overall good adherence to the prescribed exercise programme with the wearable device appearing to provide a greater impact on perceived quality of life. This appears as good news for patients and prescribers because whatever is the method, telehealth monitoring sustains positive effects for health in KTRs undergoing exercise training programs.

Exercise intervention therapy, as an approach focused on functional exercise, has gained prominence in recent years as an adjunctive treatment strategy for various surgical and non-surgical procedures, including solid organ transplantation (26). Furthermore, the application of exercise intervention programs has been investigated in the post-transplantation setting, with notable findings in different organ transplant scenarios (27). Four recent systematic reviews with meta-analysis conducted exploring exercise as an intervention in KTRs showed as it can improve kidney function, cardiopulmonary fitness, dyslipidaemia, physical performance, and quality of life in KTRs (6, 7, 28, 29). Our results are in agreement with these reviews adding an interesting insight into exercise prescription and adherence with different telehealth monitoring methods.

### 4.1 Body composition parameters

Our study did not show significant changes in BMI, waist circumference, or percentage of lean and fat mass during the monitored period. This is in line with previous findings suggesting the complex relationship between exercise and body composition in transplant and specifically KTRs (6, 28). Individual variability, different exercise modalities and potential interactions with immunosuppressive medications may contribute to the observed stability. Indeed, the only study on KTRs that showed a relevant effect of exercise training on BMI was conducted on patients with severe obesity (11).

### 4.2 Blood and urinary test

The observed improvement in HDL levels over the one-year intervention period aligns with well-established evidence linking regular exercise to favourable lipid profiles. Exercise has been consistently associated with increased HDL cholesterol, a key component of cardiovascular health, known for its role in reverse cholesterol transport and anti-inflammatory properties, not necessarily with significant reductions in total cholesterol, supporting the potential cardioprotective effects of exercise in KTRs (30). Some studies have shown a protective effect on the atherogenic risk of exercise on KTRs (31, 32) but others did not report substantial changes in the lipid profile, probably influenced by the role of immunosuppressive therapy in altering the overall lipid balance (28, 33).

The consistent maintenance of blood glucose levels within the normal range throughout the study supports the notion that exercise interventions in KTRs may contribute to glycaemic control still mitigating the potentially hyperglycaemic role of immunosuppressive therapies. The study by Morales Febles and colleagues emphasized the role of exercise in improving insulin sensitivity and glycaemic regulation in KTRs, corroborating our findings (34).

The modest decrease in creatinine and urea, although not statistically significant, suggests a potential trend toward improved kidney function with exercise. Previous literature has shown that exercise may enhance kidney blood flow and mitigate its dysfunction in various populations with kidney diseases (35). In KTRs, the evidence is conflicting, with several studies showing an improvement in kidney function markers (11, 32, 36) and as many finding no significant difference after the exercise interventions (10, 37, 38).

The reduction in inflammatory markers among participants with initially elevated levels highlights a potential anti-inflammatory effect of exercise. Chronic inflammation is a known contributor to graft dysfunction and cardiovascular complications post-transplant (39). Aerobic and resistance training at moderate intensity may exert anti-inflammatory effects in KTRs with a reduction in tumour necrosis factor alpha levels and modulating interleukin-6 blood level (38, 40).

### 4.3 Physical performance

The significant increase in absolute and relative VO_2_ peak underscores the positive impact of the exercise intervention on cardiorespiratory fitness in KTRs (6, 7, 28, 29, 41, 42). The observed benefits in cardiorespiratory fitness are crucial given their association with reduced cardiovascular risk and improved overall mortality in all patients with chronic diseases, including KTRs. Indeed, a recent exploratory analysis showed as each increase in VO_2_ peak of 1 ml/kg/min was associated with a 0.5% decrease in 7-year risk of major adverse cardiovascular events and 1% decrease in 7-year risk of mortality in KTRs (43). Moreover, our study group showed as low cardiorespiratory fitness may be considered as a modifiable predictor of long-term severe infectious events in KTRs stressing the importance of CPET in cardiovascular evaluation of this specific population (44). Finally, assessing ventilatory thresholds through CPET allows the formulation of a tailored exercise prescription aimed at improving the functional capacity of KTRs (4, 45).

Otherwise than cardiorespiratory fitness, handgrip strength did not exhibit a significant increase, confirming the heterogeneous findings on strength in KTRs from recent evidence (36, 37). The non-significant change and the small decrease in strength in the dominant upper limb between T6 and T12 may be influenced by individual variations in response to exercise and the tendency to engage in predominantly aerobic rather than strength activities when training was unsupervised.

The increase in repetitions during the 30SCT at T6, sustained at T12, suggests an improvement in muscular endurance. This finding resonates with previous studies indicating that exercise interventions positively impact functional capacity, including chair stand performance, in KTRs (37, 38, 46).

The increase in IPAQ scores at 6 months, followed by a further rise at 12 months, suggests a sustained improvement in self-reported physical activity levels. These findings align with the concurrent increase of the cardiorespiratory fitness in our study and with existing literature emphasizing the positive impact of structured exercise interventions on promoting and maintaining increased physical activity in KTRs (42).

### 4.4 Quality of life and exercise adherence

Ensuring long-term exercise adherence is one of the major issues inherent in exercise prescription (47, 48). During the first half of the study, patients were initiated into supervised exercise in our hospital gym and then monitored monthly with videocall interviews until T6. The subdivision of the initial cohort at T6 into a WG, employing continuous objective monitoring, and a VG, relying on monthly video interviews, provided valuable insights into the different monitoring approaches on exercise-related outcomes. The lack of significant differences in most parameters, except for higher PCS and MSC scores in WG, showed how the method itself does not bring a direct clinical benefit to the KTRs but leads to a better quality of life perception by the patients. Different studies indicated that wearable device-based monitoring is associated with improved health-related quality of life indices in various clinical populations (49, 50). The ability of wearables to provide real-time feedback and personalized insights may contribute to enhanced motivation and engagement in prescribed exercise regimens, translating into improved mental well-being also in KTRs (51). Objective monitoring offers advantages over self-report methods by providing accurate data on volume and intensity levels accomplished during exercise training (52, 53). This supports the growing consensus that incorporating objective monitoring into post-transplant exercise interventions enhances precision and reliability in assessing patient responses (54). On the other hand, VG reported even higher levels of physical activity. Probably, the possibility of being able to see and talk to an exercise professional on a regular basis provided more specific benefits, such as gaining information about the type of exercise practised, and any problems associated with the exercise performed with the health condition. This interaction modality belongs to the larger world of telehealth using digital interventions to improve physical fitness, which has proven its worth for various chronic diseases (55–60), including KTRs (14, 15).

**Figure 4:**
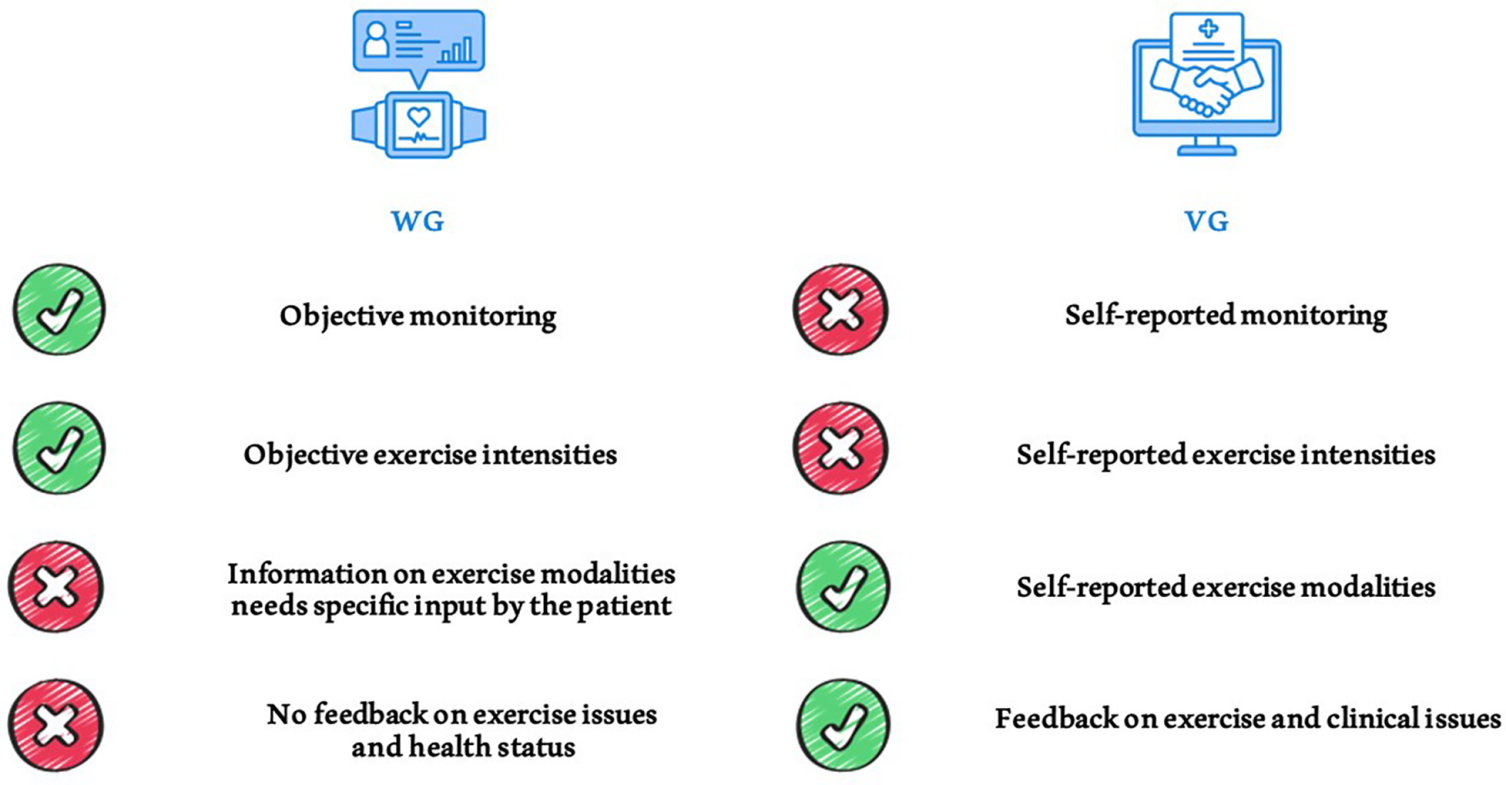
Comparison of different exercise monitoring methods used in the study. Pros and cons of using telehealth tools such as wearables and videocall interviews for monitoring physical activity and exercise training in patients.

A notable focus is the exceptional adherence observed in both groups, where most patients consistently adhered to the exercise prescription over an extended period. We used the moderate to vigorous exercise intensity model (MVPA) for quantifying adherence. A patient was considered adherent to the prescribed exercise if they engaged in at least 150 min per week of moderate-intensity physical activity or 75 min per week of high-intensity physical activity (or a combination of both), as recommended by WHO guidelines. Our patient reported more than 200 min per week in mean of moderate exercise, more than the minimum recommended. This aligns with current literature emphasizing the positive impact of health information technology on exercise adherence in KTRs, regardless of the intervention tools (61). Both videocalls and wearable devices interventions can improve self-management in KTRs, with different pros and cons (Figure 4), tracking progress and contributing to the sustained adherence observed in the study sample.

Healthcare system should have a key role in supporting KTRs to become more physically active with the application of different available tools to monitor and improve patients' adherence to therapeutic strategies, including exercise training.

## 5 Study limitations and perspectives

It is essential to acknowledge the limitations of this study, including the relatively small sample size and the potential for selection bias due to the one-year exercise program with three complete assessments. Indeed, of the initial enrolled cohort only 65% of the participants completed the intervention phase up to the final evaluation. Long-term studies with larger cohorts are needed to confirm the durability of the observed improvements and to account for potential confounding variables. A potential limitation dictated by non-regular use of the wearable by patients was minimised by the high compliance of the patients. Future research endeavours should focus on expanding the understanding of the optimal exercise prescription parameters, including intensity, duration, and modality. Additionally, investigations into the long-term impact of exercise on graft survival and transplant-related comorbidities will further inform clinical practice.

## 6 Conclusions

This study supports the clinical relevance of integrating tailored exercise training into standard kidney transplant care due to the improvement of physical fitness, cardiovascular risk factors and quality of life. Moreover, these findings underscore the importance of using telehealth monitoring methods as an incentive for maintaining good adherence to the exercise prescription in the long-term. Solutions are needed to overcome barriers to equal access to these instruments so that all KTRs can benefit from high-quality exercise interventions.

## Data Availability

The raw data supporting the conclusions of this article will be made available by the authors, without undue reservation.
